# Renal Replacement Therapy in Idiopathic Systemic Capillary Leak Syndrome: A Case Report

**DOI:** 10.7759/cureus.53982

**Published:** 2024-02-10

**Authors:** Masayuki Tokutake, Ai Nakazawa, Masafumi Ota

**Affiliations:** 1 Emergency Department, Tsugaruhoken Medical COOP Kensei Hospital, Hirosaki, JPN

**Keywords:** idiopathic systemic capillary leak syndrome, hypotension, hemoconcentration, hypoalbuminemia, renal replacement therapy, acute kidney injury, systemic capillary leak syndrome

## Abstract

Idiopathic systemic capillary leak syndrome (ISCLS) is a rare disease characterized by hypotensive shock, anasarca, hemoconcentration, and hypoalbuminemia. Despite the life-threatening course of the disease, no treatment strategy has been established. A 68-year-old man presented with hypotensive shock following a prodrome. Based on the characteristic blood test findings, ISCLS was suspected. The patient was resuscitated by administering massive amounts of fluids and inotropic and vasopressor agents. After his blood pressure had stabilized, renal replacement therapy (RRT) was promptly initiated to facilitate the removal of excess fluid, despite the presence of urine output. Typically, ISCLS has three phases: prodromal, leak, and post-leak. Diuresis should be promptly induced during the transition from the leak phase to the post-leak phase to avoid fatal complications such as pulmonary edema. We propose that in patients with ISCLS, early introduction of RRT is recommended if indicated.

## Introduction

Idiopathic systemic capillary leak syndrome (ISCLS), also known as Clarkson’s disease, is a rare disease [1]. The diagnostic criteria for ISCLS comprise three clinical manifestations: hypotension, hemoconcentration, and hypoalbuminemia, and it is diagnosed by exclusion [2]. Hypotensive shock is severe in several cases and requires massive fluid infusions for resuscitation. This often results in fatal complications such as pulmonary edema [3]. Because there are only a few reports of ISCLS, a treatment strategy is yet to be established [2]. Herein, we have described a case of ISCLS in which renal replacement therapy (RRT) was promptly administered after resuscitation in addition to massive fluid replacement to save the patient’s life without exacerbation of complications such as pulmonary edema.

## Case presentation

A 68-year-old male was referred to our hospital from a neighboring community hospital for fatigue, bilateral lower limb weakness, and palpitations for the past two days. Concerning the medical history, he presented to our hospital one year ago with complaints of fatigue and bilateral leg myalgias after an antecedent infection. The symptoms at this time had resolved spontaneously; however, the cause remained unknown. In the present occasion, there was no apparent antecedent infection, unlike past episodes. On admission, his peripheries were cold, his skin was mottled, and he had a fast and weak pulse. His blood pressure was too low to measure, his heart rate was 112 beats per minute, his respiratory rate was 32 breaths per minute, and his body temperature registered at 35.9℃. He required the administration of 10 liters per minute of oxygen through a face mask to maintain adequate peripheral oxygen saturation. Laboratory tests revealed hemoconcentration, hypoalbuminemia, and acute mixed acidosis (Table 1). He was treated with fluid resuscitation, hydrocortisone, and broad-spectrum antibiotics for hypovolemic shock or distributive shock. Despite administering 2 L of lactated Ringer’s bolus, he became unconscious and was managed with artificial ventilation. The patient was transferred to the high care unit (HCU).

**Table 1:** Laboratory results on arrival * Reference range of arterial blood PCO_2_, Partial pressure of carbon dioxide; HCO3^-^, bicarbonate; SARS-CoV-2, Severe acute respiratory syndrome coronavirus 2

Variable	Reference range	Results
Venous blood gas analysis*		
pH	7.35-7.45	7.129
PCO_2_	35.0-45.0 mmHg	65.9
HCO3^-^	22.0-28.0 mmol/L	20.9
Lactate	4-14 mg/dL	63
Complete blood cell count		
White blood cells	3,300-9,000/μL	17,400
Hemoglobin	13.5-17.5 g/dL	18.7
Hematocrit	39.7-52.4%	57.4
Platelets	14.0-34.0×10^4^/μL	7.1
Biochemistry		
Total protein	6.7-8.3 g/dL	4.0
Albumin	3.8-5.2 g/dL	1.9
Aspartate aminotransferase	10-40 U/L	19
Alanine aminotransferase	5-45 U/L	8
Lactate dehydrogenase	124-222 U/L	239
Urea nitrogen	8-20 mg/dL	45.9
Creatinine	0.61-1.04 mg/dL	1.51
Creatine kinase	60-270 U/L	185
Sodium	137-147 mEq/L	133
Potassium	3.5-5.0 mEq/L	5.3
Serum chloride	98-108 mEq/L	101
Calcium	8.4-10.4 mg/dL	6.9
C-reactive protein	0.0-0.3 mg/dL	1.67
Brain natriuretic hormone	0-18.4 pg/mL	193.3
Polymerase chain reaction for SARS-CoV-2		negative

**Figure 1:**
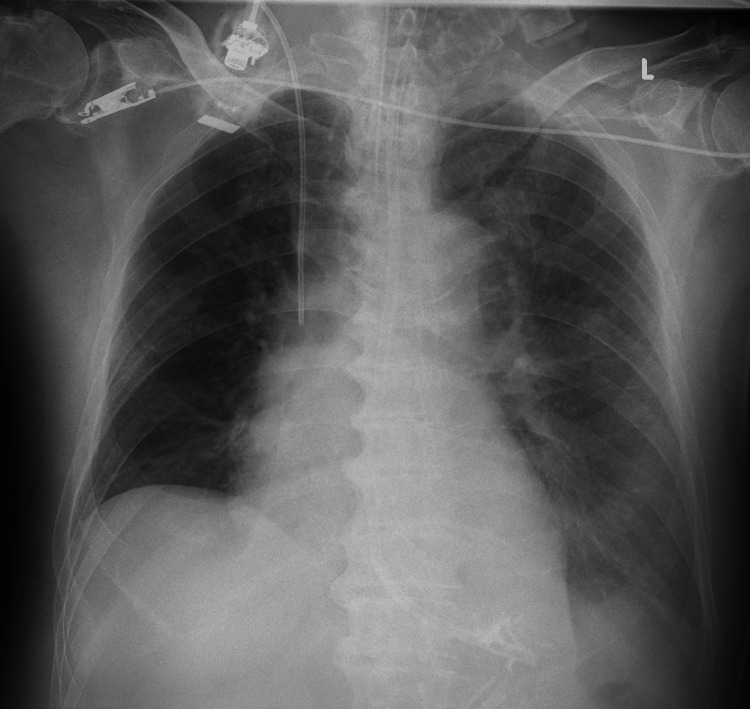
Chest X-ray showed pulmonary edema and pleural effusion on the fifth day of admission

On the first day in the HCU, the patient was administered fluid resuscitation and inotropic and vasopressor agents to maintain the blood pressure. An additional 5.5 L of Ringer’s bicarbonate solution and 1 L of isotonic albumin preparation were infused per day. Norepinephrine and vasopressin were administered at a maximum dose of 1.4 μg/kg/min and 0.03 units/min, respectively. From the next day, the fluids and inotropic and vasopressor agents were gradually tapered. On the fourth day, his intravascular volume was restored, and the inotropic and vasopressor agents were discontinued because his blood pressure had stabilized. Despite being classified as acute kidney injury (AKI) stage III based on the Acute Kidney Injury Network criteria [4], a urine output exceeding 300 mL was achieved within 2 hours following the intravenous administration of 1 mg/kg of furosemide. Having exceeded the cutoff in a furosemide stress test [5], he underwent diuretic administration initially: a continuous intravenous infusion of furosemide at a dosage of 60 mg per day, along with an intravenous infusion of tolvaptan at a dosage of 8 mg per day. On the fifth day, the patient’s fluid status was a positive balance at 13 L, central venous pressure (CVP) remained high, and anasarca was observed. The PaO_2_/FiO_2_ (P/F) ratio was 150, and chest X-ray showed pulmonary edema and pleural effusion (Figure 1).

**Figure 2:**
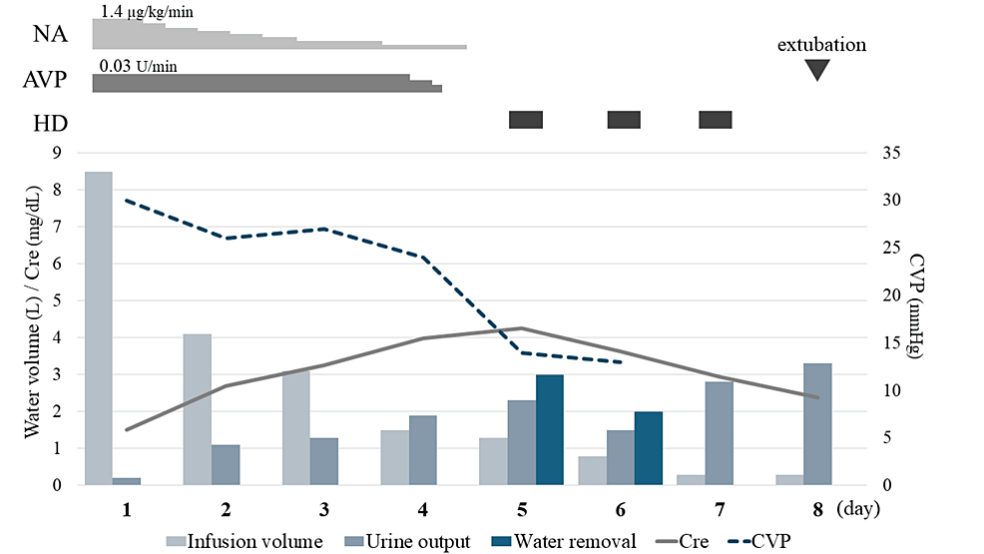
Treatment course NA, noradrenaline; AVP, arginine vasopressin; HD, hemodialysis; Cre, creatinine; CVP, central venous pressure

Because of the exacerbation of respiratory setting and image findings, the imperative for an expedited correction of body fluid volume was established. Although the equivalent diuretic dosage as the previous day was continued and urine output of 2 mL/kg/h or more was maintained, hemodialysis (HD) was introduced as RRT because of a presumed diuretic-resistant state due to renal congestion. After the introduction of HD, urine output increased, CVP decreased and respiratory status tended to improve. On the seventh day, because the fluid balance was maintained only through spontaneous urination, HD was continued for solute removal, not for water removal. On the eighth day, the P/F ratio improved to 230, and the patient was weaned from ventilator and HD. The treatment course from HCU admission to extubation is shown in Figure 2. Subsequently, he was transferred to the medical ward and discharged without neurological sequelae on the 35th day of hospitalization.

Repeated echocardiographic examinations did not reveal any signs of cardiogenic shock during the patient’s stay in the HCU. Because blood and urine cultures conducted prior to the commencement of antibiotic therapy were negative and whole-body contrast-enhanced computed tomography and diffusion-weighted magnetic resonance imaging showed no obvious focus of infection, septic shock was ruled out. The 24-hour urine protein excretion was 0.6 g, which failed to satisfy the diagnostic criteria for nephrotic syndrome. The patient was diagnosed with ISCLS based on the concurrent presence of the typical triad of hypotension requiring extensive fluid therapy, hemoconcentration, and hypoproteinemia. Subsequent serum immunofixation electrophoresis and bone marrow examinations revealed complications of IgGλ-type monoclonal gammopathy of undetermined significance, which also supported this diagnosis [6]. We considered administering a high dose of intravenous immunoglobulin based on the literature that it was effective in preventing the recurrence of ISCLS [7], but it was short in supply. Eight months after discharge, no recurrence has been observed.

## Discussion

ISCLS is a rare disease characterized by transient episodes of severe hypotensive shock and anasarca [1]. It is believed to be caused because of hemoconcentration and vascular collapse due to the extravasation of plasma. Blood tests demonstrate characteristic findings of low albumin levels despite elevated hemoglobin levels and hematocrit [3].

A typical ISCLS episode follows three phases: prodromal, leak, and post-leak [2]. In the prodromal phase, the patient exhibits a typical prodrome such as generalized weakness, fatigue, and myalgias. In the leak phase, shock and edema develop rapidly due to plasma extravasation. In the post-leak phase, which occurs several days after the leak phase, the patient rapidly recovers from circulatory failure because fluid is mobilized from the third space into the main circulation. There is no established treatment for ISCLS. Furthermore, because the complications vary depending on the phase of ISCLS, the treatment should be tailored to each phase [2,6]. During the leak phase, hypovolemic shock due to intravascular volume depletion leads to hypoperfusion-related multiorgan dysfunction, including AKI. Concurrently, hypercoagulability due to hemoconcentration and increased serum viscosity leads to thrombotic events, such as deep venous thrombosis and pulmonary embolism [6]. Patients require massive infusions of colloids and inotropic and vasopressor agents to maintain the intravascular volume. Furthermore, the efficacy of Intravenous Immunoglobulins (IVIG) administration during the leak phase has been reported [8]. In our case, IVIG was not administered as the confirmation of ISCLS diagnosis was unattainable during the acute stage. Excessive infusion resuscitation during the leak phase causes anasarca, noncardiogenic pulmonary edema, and compartment syndrome in the extremities [2]. Therefore, diuresis should be encouraged without delay during the post-leak phase [9].

RRT has been used for the treatment of AKI in patients with hypouresis and/or pulmonary edema [10-12]. In the present case, we introduced RRT early on to prevent exacerbation of pulmonary edema in the post-leak phase despite the presence of urine output because the patient had a positive balance of 13 L on the fifth day. To maintain glomerular filtration rate (GFR), it is important to maintain renal perfusion pressure which is related to mean arterial pressure and venous pressure [13]. In the leak phase of ISCLS, capillary permeability increases, and protein-rich fluid moves from the intravascular space to the interstitial space [9]. The decrease in intravascular volume leads to a decrease in mean arterial pressure, which decreases renal perfusion pressure and lowers GFR. When capillary permeability normalizes during the post-leak phase, the fluid accumulated in the interstitial space is mobilized back into the intravascular lumen [9]. At this phase, the excess intravascular fluid causes renal congestion due to increased renal venous pressure, which decreases renal perfusion pressure and lowers GFR. It is also at this stage that pulmonary edema, similarly caused by elevated venous pressure, occurs. The treatment of ISCLS poses a dilemma between requiring massive fluid infusions for resuscitation and maintaining renal function during the leak phase and life-threatening complications such as pulmonary edema caused by excess fluids in the post-leak phase. If renal dysfunction is progressing due to renal congestion by excess fluids, correction of body fluid volume will improve renal function. The prompt introduction of RRT has the effect of rapidly correcting fluid volume, which promotes self-urinary excretion and further improves renal function. As a result, fatal complications such as pulmonary edema can be avoided.

## Conclusions

The main treatment for idiopathic systemic capillary leak syndrome (ISCLS) is supportive care, and the three phases of the clinical course should be considered. During the leak phase, sufficient fluid replacement and inotropic and vasopressor agents are required. However, in the post-leak phase, aggressive diuresis is required to prevent complications such as pulmonary edema. A decrease in the fluid and cardiovascular agent requirement indicates a transition from the leak phase to the post-leak phase. At this time, early hemodialysis may aid in promoting volume removal and avoiding complications by reducing renal congestion. Further accumulation of more cases is required to establish a treatment strategy for ISCLS that are associated with fewer complications.

## References

[1] Clarkson B, Thompson D, Horwith M, Luckey EH (1960). Cyclical edema and shock due to increased capillary permeability. Am J Med.

[2] Druey KM, Greipp PR (2010). Narrative review: Clarkson disease-systemic capillary leak syndrome. Ann Intern Med.

[3] Kapoor P, Greipp PT, Schaefer EW (2010). Idiopathic systemic capillary leak syndrome (Clarkson's disease): the Mayo Clinic experience. Mayo Clin Proc.

[4] Mehta RL, Kellum JA, Shah SV, Molitoris BA, Ronco C, Warnock DG, Levin A (2007). Acute Kidney Injury Network: report of an initiative to improve outcomes in acute kidney injury. Crit Care.

[5] Chawla LS, Davison DL, Brasha-Mitchell E (2013). Development and standardization of a furosemide stress test to predict the severity of acute kidney injury. Crit Care.

[6] Druey KM, Parikh SM (2017). Idiopathic systemic capillary leak syndrome (Clarkson disease). J Allergy Clin Immunol.

[7] Xie Z, Chan EC, Long LM, Nelson C, Druey KM (2015). High-dose intravenous immunoglobulin therapy for systemic capillary leak syndrome (Clarkson disease). Am J Med.

[8] Lambert M, Launay D, Hachulla E (2008). High-dose intravenous immunoglobulins dramatically reverse systemic capillary leak syndrome. Crit Care Med.

[9] Siddall E, Khatri M, Radhakrishnan J (2017). Capillary leak syndrome: etiologies, pathophysiology, and management. Kidney Int.

[10] Tanabe M, Hikone M, Sugiyama K, Hamabe Y (2022). Systemic capillary leak syndrome requiring fasciotomy for limb compartment syndrome: A case report and literature review. Acute Med Surg.

[11] Fukuda M, Nabeta M, Hirayu N, Kannae M, Takasu O (2023). Systemic capillary leak syndrome induced by influenza type A infection: a case report. Cureus.

[12] Naito S, Yamaguchi H, Hagino N (2023). Systemic capillary leak syndrome as a rare, potentially fatal complication of COVID-19: a case report and literature review. Cureus.

[13] Kopitko C, Medve L, Gondos T (2018). Renoprotective postoperative monitoring: what is the best method for computing renal perfusion pressure? An observational, prospective, multicentre study. Nephron.

